# Redescription of *Stenolophus thoracicus* Casey (Coleoptera, Carabidae, Harpalini), a valid species

**DOI:** 10.3897/zookeys.53.470

**Published:** 2010-08-27

**Authors:** Yves Bousquet, Peter W. Messer

**Affiliations:** 1 Agriculture and Agri-Food Canada, Central Experimental Farm, Ottawa, Ontario K1A 0C6, Canada; 2 4315 W River Lake Dr., Mequon, Wisconsin 53092, U.S.A.

**Keywords:** Coleoptera, Carabidae, Stenolophus, Agonoleptus, Nearctic, taxonomy

## Abstract

Stenolophus thoracicus Casey is revalidated. The species is redescribed based on a study of the syntypes and of several conspecific specimens from eastern North America. The species differs from the other eastern species of the subgenus Agonoleptus in having the metasternum shorter and the wings reduced to tiny stubs. The dorsal habitus and median lobe of the aedeagus, along with the structures of the internal sac, are illustrated.

## Introduction

Lindroth (1968: 921), in his comprehensive monograph of the carabids of Canada and Alaska, synonymized Stenolophus thoracicus Casey with Stenolophus conjunctus (Say) pointing out, however, that the microsculpture on the pronotum of the female “type” was considerably stronger than normal. A study of Casey’s syntypes revealed that the specimens are in fact specifically distinct from those of Stenolophus conjunctus. The purpose of this paper is to redescribe Stenolophus thoracicus and to discuss the structural differences between Stenolophus thoracicus and the other eastern species of the subgenus Agonoleptus Casey.

The holdings of the following collections were studied: Buffalo Museum of Science, New York (BMSC), California Academy of Sciences (CAS), National Collection of Insects, Arachnids and Nematodes (CNC), Field Museum of Natural History, Chicago (FMNH), Los Angeles County Museum of Natural History (LACM), Museum of Comparative Zoology (MCZ), Michigan State University Collection (MSUC), North Dakota State University (NDSU), National Museum of Natural History (USNM), University of Wisconsin-Madison Insect Research Collection (WIRC), and the collection of Peter W. Messer (PWM). No examples of Stenolophus thoracicus were observed in material from University of California at Berkely, Milwaukee Public Museum, Mississippi State University, and Washington State University.

## Stenolophus thoracicus

Casey, 1914

Stenolophus thoracicus Casey, 1914: 282. Type locality: «S[ain]t Louis, Missouri» (original citation for the lectotype).

### Type material.

Lectotype (♂), designated by Lindroth (1975: 143), in USNM labelled: “Mo / ♂ / Casey bequest 1925 / thoracicus Paratype USNM 48052 / Lectotype thoracicus Csy by C.H. Lindroth.” Casey’s collection includes six other specimens under the name Stenolophus thoracicus (one ♀, five ♂), each labelled later as “paratype.” The unit tray bears a label “type missing Lindroth 73.” It seems that the Casey’s collection included another female specimen, labelled as “type,” that Lindroth studied before 1968.

**Figure 1:**
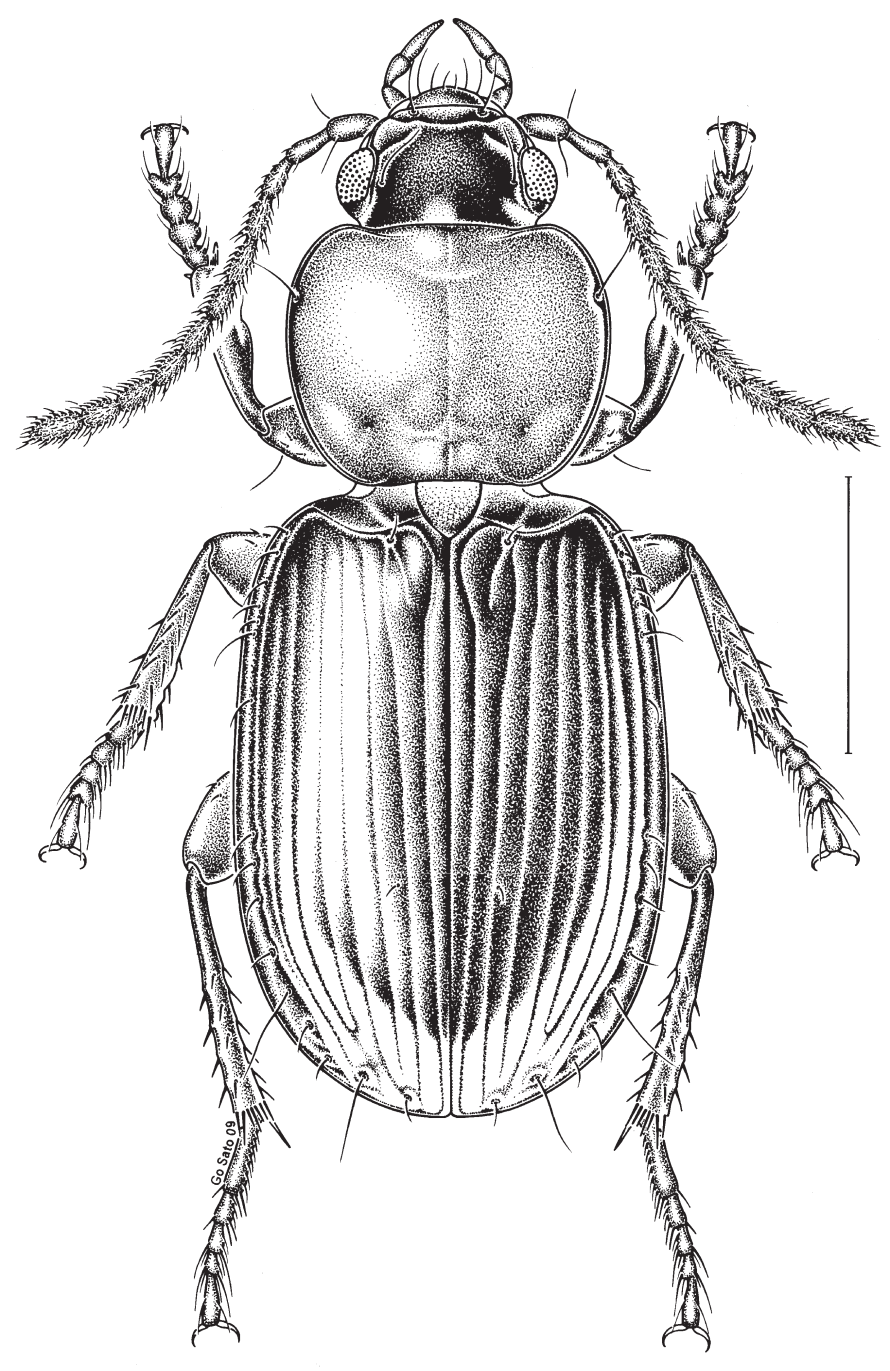
Habitus (dorsal view) of Stenolophus thoracicus.

### Description^1^.

Coloration. Clypeus and labrum reddish; frons reddish-brown to reddish-black; antennomeres 1 and 2 yellow, antennomeres 3–11 slightly darker, brownish-yellow; pronotum entirely yellow to reddish-yellow; elytra reddish-brown to reddish-black, with base, intervals 1 and lateral margins paler, more or less yellowish to reddish; elytral epipleura yellow; legs entirely yellow. Microsculpture. Frons with isodiametric meshes, meshes indistinct or almost so in the male, faint but distinct in the female; pronotum with linear microlines, microlines indistinct or almost so in the male, distinct in the female; elytra with markedly transverse meshes, meshes well impressed in both sexes. Head. Clypeo-ocular line evident, complete (i.e., reaching medial edge of eye). Pronotum. Proportionally wide, LP/WP = 0.73–0.79 (mean = 0.76; n= 10); maximum width clearly anterior to middle; anterior angle markedly protruding; basal impression shallow, almost indistinct in some specimens; basal bead reaching just beyond level of basal impression. Elytra. Striae impressed, shallow but deeper toward apex, impunctate; intervals flat. Thorax (ventral side). Metasternum, short, length behind mesocoxa about 0.7 that of metacoxa along same line. Male genitalia. Median lobe with apex hooked; internal sac with two large U-shaped sclerotized structures and a small “scaly body” near middle.

Apparent body length: 3.5–4.1 mm.

**Figure 2:**
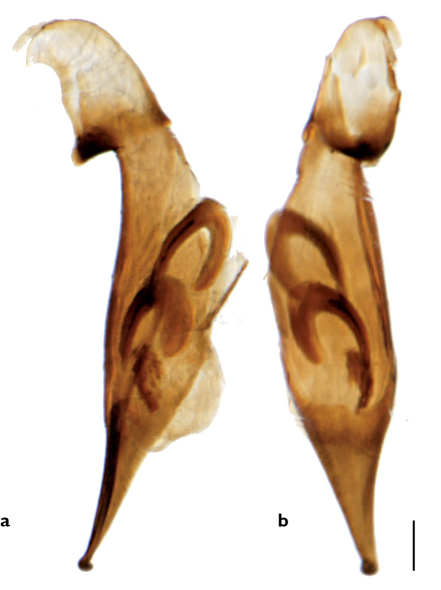
Median lobe of aedeagus of Stenolophus thoracicus. **a** left lateral view **b** ventral view.

### Geographical distribution.

**Figure 3:**
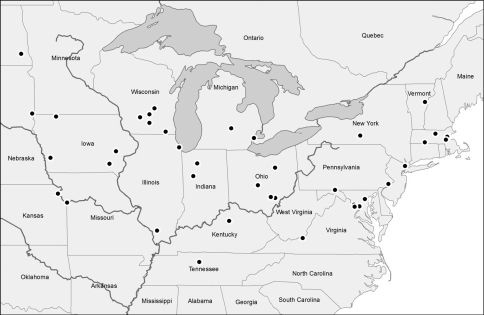
Collection localities for Stenolophus thoracicus.

This species ranges from east-central Vermont to southeastern North Dakota, southwardly to northeastern Kansas, Tennessee, and northeastern Virginia (Fig. 3). We have seen specimens from the following localities. District of Columbia. Woodridge, 14.IV.1898 (1, USNM). Illinois. Chicago, Cook Co., IV.1980, Blackwelder (1, WIRC). Pine Hills Field Station, Union Co., 15–22.V.1967, J.M. Campbell (3, CNC). Indiana. Pulaski Co., 14.V.1977, N.M. Downie (1, FMNH). Tippecanoe Co., 3.VII.1961, N.M. Downie (1, FMNH). Iowa. “Ia.” (3, USNM). Jefferson Co. (1, USNM). Pottawattamie Co. (2, USNM). Iowa City, Johnson Co., 1.X.1917, L. Buchanan (1, USNM). Okoboji, Dickinson Co., VIII (2, USNM). Kansas. Atchison, Atchison Co., 25.IV. (1, USNM). Kentucky. Frankfort, Franklin Co., 16.IV.1892 (1, USNM). Maryland. Baltimore, 16.V.1909, F.E. Blaisdell (1, LACM). Massachusetts. Cambridge, Middlesex Co., 18.III.1974 (1, MCZ). Waverly, Middlesex Co. (1, USNM). Springfield, Hampden Co., G. Dimmock (1, MCZ). Dover, Norfolk Co., 19.IV.1904, F.C. Bowditch (1, MCZ). Dover, Norfolk Co., 4.VI.1908, A. P. Morse (1, MCZ). Wachusett, Worcester Co., 19.IV.1906, Perry Gardner Bolster (1, MCZ). Bolton, Worcester Co., 13.IV.2010, T. Murray (1, PWM). Michigan. Rose Lake Wildlife Experiment Station, Clinton Co., 18.XII.1971, D.K. Young (1, WIRC). Detroit, Wayne Co. (1, USNM). Missouri. “Mo” (2, USNM). Kansas City, 26.IV.1898 (1, USNM). New Jersey. “N.J.,” R. Hopping (1, CAS). Tenafly, Bergen Co., 17.III.1917, F.M. Schott (1, CNC). New York. “N.Y.” Schaupp (1, BMSC). Ithaca, Tompkins Co., 25.VIII.1911, Van Dyke (1, CAS). North Dakota. Mirror Pool, Richland Co., T135N-R52W-Sec 8, NE ¼, 27.V.1966, Gordon & Aarhus (1, NDSU). Ohio. Wayne Co., 1.V.1938 (2, MSUC). W’ Loo Twn., Athens Co., 6.V.1936, W. Stehr (1, MSUC). Canaan Twn., Athens Co., 24.III.1939, W.C. Stehr (1, MUSC). Carbondale, Athens Co., 21.V.1938, 8.IV.1948, W.C. Stehr (2, MSUC). Columbus, Franklin Co., 23.IV.1929, R.T. Everly (2, MSUC). Pennsylvania. 6 km SW Buck Valley at Sideling Hill Creek, 39°44'N, 78°21'W, 10.X.1992, W.E. Steiner & J.M. Swearingen (1, USNM) [shale barren slope]. Frankford, Philadelphia Co., A. Schmidt (1, USNM). South Dakota. [East] Sioux Falls, Minnehaha Co., 25.IV.1967, V.M. Kirk (1, USNM) [sod, rock]. Tennessee. Nashville, Davidson Co. (1, USNM). Vermont. Topsham, Orange Co., 27.X.2008, T. Murray (1, PWM). Virginia. “Va”, 29.V.1881 (1, USNM). Great Falls, Fairfax Co., 29.X., Banks (2, MCZ). Vienna, Fairfax Co., 2.X.1932 (1, USNM). Fairfax Co., 21.IX.1930, A. Nicolay (1, USNM). Fairfax Co., 4.VI.1972, R. Gordon & A. Cushman (1, USNM). 9 km N Mountain Lake, Wind Rocks, Giles Co., 19.VIII.1984, W. Steiner & J. Hill (1, USNM). Wisconsin. “Wis” E. Chope (1, FMNH). Mud Lake Nat. Site, Columbia Co., 21–27.V.1996 (1, WIRC). Madison, Dane Co., 27.IV.1910, J.G. Sanders (1, WIRC). Nevin Marsh, Dane Co., 12.VI.1974, D.T. Bach (1, WIRC). Green Lake Co., 22.IV.2000, C. Buss (1, WIRC). Hemlock Draw, Sauk Co., 6.V.2007, J.P. Gruber (1, PWM). Springfield Nat. Site, Walworth Co., 8–15.VII.1996 (3, WIRC).

### Habitat.

No information is available about the habitat requirements of the species.

### Wing condition.

Nine specimens were dissected and their wings were reduced to tiny stubs. Considering the size of the metasternum, the species is very likely constantly brachypterous.

### Note.

Stenolophus thoracicus belongs to the subgenus Agonoleptus Casey which includes six other species-group taxa: Stenolophus conjunctus (Say), Stenolophus rotundicollis (Haldeman), Stenolophus rotundatus LeConte, Stenolophus parviceps (Casey), Stenolophus unicolor Dejean, and Stenolophus unicolor dolosus Casey. The last three-named taxa are found in southwestern United States and are not further dealt with. The other taxa occur sympatrically with Stenolophus thoracicus east of the Rocky Mountains, although one of them, Stenolophus conjunctus, also extends to the West Coast.

Adults of Stenolophus thoracicus differ most notably from those of the three other eastern species of the subgenus in having the metasternum shorter and the wings highly reduced. For comparative purpose, the ratio of the metasternal length behind the mesocoxa and the metacoxal length measured along the same line varies between 1.0 and 1.2 in Stenolophus conjunctus, Stenolophus rotundicollis, and Stenolophus rotundatus. In addition, the pronotum is more narrowed posteriorly on average.

Adults of Stenolophus conjunctus differ from those of Stenolophus thoracicus also in having the microsculpture on pronotum indistinct in both sexes and the elytral microsculpture less impressed. The apex of the median lobe of the aedeagus is proportionally longer, not hooked, and the internal sac has two slightly curved sclerotized structures (see Lindroth 1968: Fig. 446a).

Adults of Stenolophus rotundicollis differ from those of Stenolophus thoracicus also in having the pronotum darker, brownish-red to reddish-brown (except narrowly around the edges) and the microsculpture on pronotum and elytra more deeply impressed. The apex of the median lobe is hooked but the internal sac contains two small U-shaped and one slender, straight sclerotized structures (see Lindroth 1968: Fig. 446c).

Adults of Stenolophus rotundatus differ from those of Stenolophus thoracicus also in having the pronotum darker, reddish-brown to piceous (except for anterior and posterior margins), and more convex, the clypeo-ocular line clearly more deeply impressed, the anterior angles of the pronotum less protruding, the medial elytral striae deeper, and the pronotum and elytra without microsculpture. The apex of the median lobe is hooked, more bluntly so than in Stenolophus thoracicus and Stenolophus rotundicollis, and the internal sac has one large U-shaped and one straight sclerotized structures (see Lindroth 1968: Fig. 446b).

### Key to eastern species of Stenolophus, subgenus Agonoleptus

**Table:** 

1.	Metasternum short, ratio of metasternal length behind mesocoxa and metacoxal length measured along the same line about 0.7	Stenolophus thoracicus Casey
–	Metasternum longer, ratio of metasternal length behind mesocoxa and metacoxal length measured along the same line 1.0 to 1.2	2
2.	Pronotum paler, reddish (in some specimens with a darkened central cloud), paler than head. Apex of median lobe not hooked	Stenolophus conjunctus (Say)
–	Pronotum darker, reddish-brown to piceous (except narrowly around edges), not clearly paler than head. Apex of median lobe hooked	3
3.	Pronotum and elytra without microsculpture	Stenolophus rotundatus LeConte
–	Pronotum and elytra with well-impressed microsculpture meshes	Stenolophus rotundicollis (Haldeman)

## Supplementary Material

XML Treatment for Stenolophus thoracicus

## Footnotes

For character states of the subgenus Agonoleptus, see Lindroth (1968: 921), as “the conjunctus group
